# An ambient assisted living architecture for hospital at home coupled with a process-oriented perspective

**DOI:** 10.1007/s12652-022-04388-6

**Published:** 2022-09-21

**Authors:** Emilio Sulis, Ilaria Angela Amantea, Marco Aldinucci, Guido Boella, Renata Marinello, Marco Grosso, Paolo Platter, Serena Ambrosini

**Affiliations:** 1 Computer Science Department, University of Turin, Corso Svizzera 185, 10149 Turin, Italy; 2 City of Health and Science, Turin, Italy; 3 AgileLab s.r.l., Turin, Italy; 4 ALTEN Italia s.p.a., Turin, Italy

**Keywords:** System architectures, Integration and modeling, Business Process Management and Integration, Healthcare, Services Integration Framework, Interoperability

## Abstract

The growing number of next-generation applications offers a relevant opportunity for healthcare services, generating an urgent need for architectures for systems integration. Moreover, the huge amount of stored information related to events can be explored by adopting a process-oriented perspective. This paper discusses an Ambient Assisted Living healthcare architecture to manage hospital home-care services. The proposed solution relies on adopting an event manager to integrate sources ranging from personal devices to web-based applications. Data are processed on a federated cloud platform offering computing infrastructure and storage resources to improve scientific research. In a second step, a business process analysis of telehealth and telemedicine applications is considered. An initial study explored the business process flow to capture the main sequences of tasks, activities, events. This step paves the way for the integration of process mining techniques to compliance monitoring in an AAL architecture framework.

## Introduction

The increasing aging of the population led to growing attention to elderly and fragile people’s assistance needs. The advent of the Internet and new information technologies immediately impacted healthcare systems, providing hospital management improvements. Several technological solutions can be applied to improve many patients’ quality of life, meeting social and medical needs. In this context, Ambient Assisted Living (AAL) is an emerging multidisciplinary research field exploiting new technologies in personal healthcare and e-Health systems combining the concepts of *ambient intelligence* and *assisted living*  (Memon et al. 2014; Calvaresi et al. 2017). AAL studies’ goal concerns monitoring services, patients’ location, recognition of activities, data collection, and behavior detection (Rashidi and Mihailidis 2013). Assistive technologies focus on patient-centered care concepts worldwide recognized as an essential dimension for the quality of care and the so-called patient empowerment. A recent literature review on the topic reveals how about half of the articles concern technology applications to different knowledge areas of health, such as patient education or medical information management (Calvillo et al. 2015). In particular, healthcare services started focusing on patient-centered approaches in the context of technology-driven ubiquitous computing. In contrast, some authors emphasized the need for a citizen-centered design approach for future cities (Streitz 2019). The present research proposes a wide framework to realize a healthcare service-oriented architecture for AAL: the system includes smart devices, an ambient-assisted intelligence framework, a computing center to manage data for Machine Learning (ML) and Artificial Intelligence (AI) applications. In addition, the framework also introduces a healthcare process analysis at the organization level.

The adoption of ICT solutions in personal healthcare and e-Health systems raised data management issues to ensure secure, meaningful data exchange between disparate technologies. Healthcare AAL applications are mostly devices used in complex socio-technical systems that require communication, collaboration, and coordination between many actors (doctors, nurses, patients, social workers, administration, caregivers, etc.). One of the main challenges is to guarantee system integration and interoperability of several different sources of next-generation AAL healthcare applications (Islam et al. 2015; Catarinucci et al. 2015). The integration of information for data and process analysis requires the adoption of interoperable systems and architectures (Yin et al. 2016). Moreover, healthcare data can be processed at the hospital and remotely, as discussed in Zhang et al. (2017), by transferring information such as electrocardiogram data (ECG) or managing remote communications with patients, e.g., teleassistance, telemonitoring, conversational agent. We opted for a secure service architecture capable of collecting data in real-time (data ingestion module based on open-source Apache Kafka platform) to perform batch operations on stored data, aggregating data sources by considering different time frequencies (data preparation) and providing functionalities exposed by an Application Programming Interface (API).

In addition to the AAL architecture concerning the data integration framework, it is relevant to consider the organization’s management perspective. An exam of the whole system involves the discipline of Business Process Management (BPM) (Dumas et al. 2018) that is currently focused on the intersection between business process analysis and data science. Information collected from different devices is stored in Process-Aware Information Systems (PAIS) (Dumas et al. 2005). A set of techniques in the field, i.e., Process Mining (PM) (Huser 2012), supports the analysis of business processes based on information stored in event logs [recently defined with a standard format, e.g., eXtensible Event Stream (Acampora et al. 2017)].

As a case study, we focus on the home hospitalization service provided by the main hospital of the “City of Health and Science” in Turin (Italy), one of the largest public health hubs in Europe with 2500 beds and more than 11,000 employees. This innovative service concerns patients’ assistance at their homes, providing them medical treatments while remaining in charge of the public hospital. Adopting an AAL architecture is relevant to managing new technological solutions for hospitalization at home, involving several small and medium-sized enterprises and two large enterprises.

The paper’s remainder is structured as follows: Sect. 2 introduces the background, related work, and the case study. Section 3 describes the platform architecture to integrate home-care applications and services, while Sect. 4 presents business process analysis in healthcare. Finally, Sect. 5 concludes the paper.

## Background

### Healthcare

AAL contributes to improving the health conditions of the population through technological solutions, products, and services having health-related outcomes in the context of the active aging paradigm, whereas everyone might live a healthy, autonomous, high-quality, and independent life (Queirós et al. 2017). Healthcare services increasingly focus on elderly and fragile people. In contrast, both the deterioration of the physical and mental abilities and the great amount of time they spend at home make it convenient to adopt technology solutions from the Internet of Things (IoT) or Internet and Communications Technology (ICT) to enhance the quality of life (Van Grootven and van Achterberg 2019). Technology solutions in AAL largely support the need for caregiving, home assistance, rehabilitation, and physical assistance (Amantea et al. 2019; Shirali et al. 2020).

A recent review of healthcare frameworks, platforms, standards of the AAL field underlines the existing gap among the requirements of real-world AAL system scenarios and the capabilities of currently available solutions and enabling technologies (Memon et al. 2014). Our work tries to overcome the issue by adopting technological solutions already developed (not in the prototype phase) by different companies operating in the field, focusing on system integration and interoperability according to the experience of a three year project, as well as a High-Performance Computing (HPC) service which is well-established for research purposes and proposed in the current framework.

### Architecture

The diversity and novelty of AAL systems engineering result from the lack of a unified model and a standardized architecture to implement and pursue while the construction of such systems (El Murabet et al. 2020). In healthcare, ICT solutions mainly concern personal wellbeing and telehealth systems developed for adaptive and anticipatory requirements. These systems necessitate high integration to achieve interoperability, usability, security, and accuracy.

The need to respect a highly interoperable approach has already focused on designing a flexible and scalable system with high performance (Chen et al. 2008; Gaynor et al. 2014). A recent literature review of the AAL field focused on healthcare frameworks, platforms, standards, and quality attributes. The most frequently addressed topic involves medical devices in healthcare systems processing citizens’ vital signs. A relevant issue concerns integrated system-of-systems, whereas the architecture defines the distribution and relationship among the AAL systems, subsystems, and components. Authors argued that “the conceptual frameworks, platforms, and architectures provide guidelines for the software architects and developers to understand the requirements of AAL systems. However, only a few of those have produced sustainable systems. Most frameworks focus only on a few aspects, ignoring the entire AAL system’s requirements as seen from different stakeholder and design perspectives. As a result, most of the reviewed frameworks produce partial solutions only that cannot support full-fledged solutions ready for real-world deployment” (Memon et al. 2014).

Several healthcare platforms and architectures have been proposed for IoT in recent years (Maskeliunas et al. 2019; Fuhrer and Guinard 2006; Catarinucci et al. 2015; Redondi et al. 2013; Hanke et al. 2011). As examples, we mention here two of the most widely adopted existing AAL architectures: FIWARE and UniversAAL. FIWARE is an open middleware platform for the development of interoperable intelligent solutions^1^ (Cirillo et al. 2019) providing public, royalty-free API specifications and interoperable protocols for creating new Internet services and applications. The FIWARE initiative adopts a very basic set of standards for collecting, managing, and publishing information about the context information. Moreover, reference open-source implementations of its components are freely available. UniversAAL is an open solution framework for connecting device drivers and applications that can simplify the development of AAL services. The framework allows applications to interoperate with each other (Stengler et al. 2015) integrating new services and deployment solutions. The middleware allows communication between different nodes using the same protocol stack. We provide some discussion points of these topics in Sect. 3.7.

This work concerns the real implementation of a healthcare AAL system, involving different companies and fully adopted by patients and hospital staff within an EU-funded research project as a case study. Concerning existing AAL prototypes and research demonstrators, it seems to help distinguish among the following five types of devices: mobile devices, smart artifacts, wearables, implants, and robots (Röcker 2011). We mostly deal with the first three types of tools in the context of telemedicine services (Peruzzini and Germani 2015). Finally, data collected in the platform can be processed with data mining and ML techniques as well as with a process-oriented analysis (Di Leva and Sulis 2017).

### AAL and process mining

In the framework of organization studies, recent efforts focused on analyzing, managing, and improving firms’ end-to-end processes. The goal of every business process analysis is to achieve three main outcomes: (1) clarity on strategic direction, (2) alignment of the firm’s resources, and (3) increased discipline in daily operations. A business process can be defined as a set of one or more linked events, activities, and decision points involving several actors and objects, collectively realizing a policy goal. Modeling is the activity involved in representing the business process of an organization. A standard modeling language is the Business Process Model and Notation (BPMN) (Aagesen and Krogstie 2015), which has proven easy to understand for hospital staff and other stakeholders involved in HHS services. Another typical representation of business processes is the Direct-Follower Graph (DFG) (Leemans et al. 2019). Section 4 introduces some examples precisely using these two types of modeling languages.

In medicine, one of the important aspects to be investigated is the organization of healthcare processes considering a systemic approach. BPM combines (medical) data science and management studies to perform healthcare process improvement (Reichert 2011), modeling and simulation (Sulis and Di Leva 2017) for decision making (Fernández-Llatas and García-Gómez 2015). Moreover, BPM includes several methods to manage business processes from real data, e.g., event logs. Most existing approaches include techniques for discovering, measuring, optimizing, and automating healthcare processes (Mans et al. 2015). Processes can be structured and repeatable, as well as unstructured and variable. The intersection of data mining and business process analysis is at the heart of the PM discipline, which applies in many areas, including healthcare (Rojas et al. 2016). PM aims to discover and measure processes by adopting automated event records in an automated manner (Amantea et al. 2020; Fernández-Llatas et al. 2013). Three main approaches in PM are: (i) “process discovery” which aims to automatically discover a process diagram from the actual event logs data; (ii) “conformance checking” which explores the differences between the current process executions (recorded in the event logs) and the actual ones; (iii) “enhancement” which aims to identify the improvement of business processes (van der Aalst 2016).

Every business process modeling activity usually starts exploring PAIS data by adopting standard modeling languages. The workflow analysis facilitates the detection of inefficiencies, bottlenecks, constraints, and risks (Suriadi et al. 2014). The goal is to obtain process improvement with the optimization of time, resources, and costs. The healthcare process model must be verified and validated by system experts, resulting in the so-called As-Is model. PM can also extract insights from event logs regarding individual behavior in smart home environments (Tax et al. 2017; Dogan et al. 2019). Habit processing from a vast amount of real-time data obtained through the Internet of Things (IoT) contributes to both AAL solutions and human behavior modeling (Lull et al. 2021). The provision of quality hospital services depends on the suitable and efficient execution of business processes. In particular, healthcare processes aim to diagnose, treat, and prevent any diseases from improving a patient’s health. These processes are supported by clinical and non-clinical activities, executed by different types of resources (physicians, nurses, technical specialists, clerks), visits and can vary from one organization to another. It is known that healthcare processes are highly dynamic, complex, ad-hoc, and increasingly multidisciplinary, having a high impact on patients’ quality of life. These features make them interesting to analyze and improve. Healthcare processes improvement presents several challenges: there is always the need to reduce the cost of services and improve capabilities to meet the demand, to reduce patients’ waiting times, to improve resource productivity, and to increase processes transparency. PM techniques can be successfully used in analyzing both clinical and administrative processes (Mans et al. 2012), as well as preserving privacy in the healthcare environment (Pika et al. 2019). Section 4.3 introduces a PM experiment from data collected in the current framework by using a Process Discovery algorithm from PM4PY library.^2^ (Berti et al. 2019)

### Data protection and privacy ethical guidelines

Nowadays an AAL project must consider privacy protection and data security issues. To ensure the protection of personal data of both patients and healthcare workers in IoT solutions, the European Regulation 2016/679, General Data Protection Regulation (GDPR) provides the EU privacy model for EU citizens. The GDPR is a European Union Regulation on personal data processing and privacy, adopted on April 27, 2016, published in the Official Journal of the European Union on May 4, 2016. The EU privacy rules include a set of individuals’ rights, data protection principles, and legal requirements.

The regulation provides guidelines for organizations on how to manage information about the individuals they interact with. Our project considers event logs files regarding human behavior from different types of sources in an IoT system. Notably, GDPR encourages organizations to consider privacy throughout the development process, which also applies to the design of AAL systems. For example, to design GDPR-compliant systems, several privacy design patterns must be considered, i.e. minimize, hide, separate, aggregate, inform, control, enforce, and demonstrate (Michael et al. 2019).

To describe privacy and ethical concerns, we mention here some key concepts. The GDPR introduces a number of figures and procedures of which we provide two examples. First of all, among the new relevant figures are the external data controller, as well as the Data Protection Officer (DPO). The DPO is a professional who has a corporate role with legal, IT, risk management and process analysis functions. The main responsibility is to assess the management and protection of personal data processing. In fact, a relevant effort concerns compliance with European and national privacy regulations. Second, the Data Protection Impact Assessment (DPIA) is a procedure provided for in Article 35 of the regulation. It describes a data processing operation to assess its necessity and proportionality, as well as the associated risks, in order to prepare appropriate measures to address them. The DPIA is an important tool in terms of accountability. The focus is on user privacy issues, also considering the 7 core principles (Cavoukian 2012), as well as a set of fair information practices. The proposed architecture addressing both privacy issues and more generally ethical concerns is treated in Sect. 3.5.2.

### Case study

Our AAL case study focuses on the Hospital at Home Service (HHS) operating in the “City of Health and Science” in Turin. HHS patients are considered public hospital patients, and all services are provided as a public service, which retains legal and financial responsibility for care. A hospital team organizes a daily visit to the patients’ domicile, where medical equipment comes directly from the hospital. The activities are defined based on the different patients’ needs. The HHS management of selected acute patients with different conditions (cardiac, respiratory, neurologic, and hematological) is feasible and non-inferior in terms of clinical outcomes than traditional in-patient management, with fewer complications, return and emergency department visits, with an improved quality of life (Isaia et al. 2010). The integrated care service team is multidisciplinary by including four geriatric doctors, two geriatric students, 14 nurses (including a nursing coordinator and a patient acceptance manager), one counselor, one social worker, four part-time physiotherapists. Patients are visited daily by medical or nursing staff, either jointly or by at least one of these two professionals. According to the available resources, each patient has their therapeutic programs as defined by daily meetings according to the clinical trend (Sulis et al. 2019b).

Within the context of an EU-funded research project (CANP)^3^ several IoT technologies for Telassistance and Telemedicine have been integrated for the treatments of HHS patients. The project involved the University of Turin (UNITO) and several industrial partners. The hospital has created a group to administer clinical trials as the Scientific Research Organization (SRO). The AAL architecture we propose relies on a general framework providing the following services integrated into an overall design:

- i. a telemedicine platform that integrates electromedical devices such as pulse oximeter, electrocardiogram;
- ii. a platform for the management of pharmacological therapies and the control of treatment compliance;
- iii. a platform for the management of clinical trials, the enrolment of patients in SROs, the collection of questionnaires and data from periodic follow-up visits;
- iv. a solution for language rehabilitation of patients with cognitive problems;
- v. an application that integrates a voice assistant with specialized skills to support patients and caregivers during therapies or biologist sample collection;
- vi. a solution for monitoring physical activity and falls, including wearable device;
- vii. an advanced telehealth system for distance training, care, and treatment of patients suffering from chronic diseases.

**Fig. 1:**
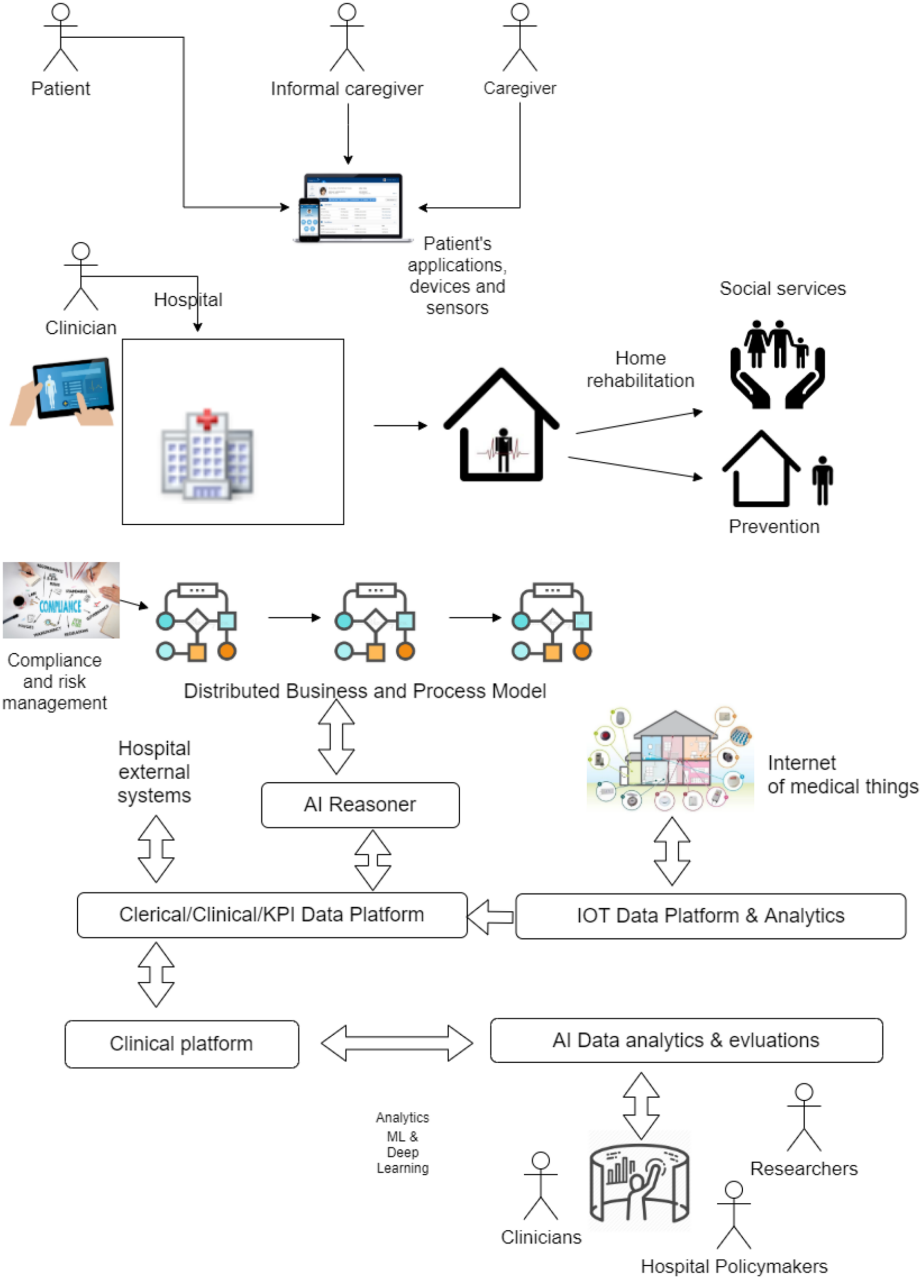
The AAL general framework about Hospital at Home Services

The general AAL framework considered in this work (see Fig. 1) aims to ensure security and privacy by design, data protection, safety, security, and trust in the resulting system and service delivery inside and outside the home and the hospital (henceforth, we refer to the CANP architecture). The following Sections describe the main parts of the architecture and the initial undergoing business process analysis.

In 2018 the HHS cared for 490 patients (mean age 84 years, range 19–104), in 46% of cases directly admitted from the emergency department, with a mean of 20 patients per day. The average length of stay is about 15 days. Most of the discharges are possible at home with or without activation of other integrated home care services (81.2%), while other patients die at home (11.8%) and are acutely transferred to the hospital (6.9%). The professional experience of the staff and critical clinical studies described the advantages in terms of clinical improvement, reduction of the complications, reduction of the re-entry in the hospital, the impact on the quality of patient’s life, as well as a reduction of the costs (i.e., from 400 to 155 Euro/ day on average).

## Platform architecture

### The general AAL architecture

The AAL architecture implies the adoption of a set of heterogeneous ICT systems with distinct functional features covering different services related to home hospitalization, such as telemedicine (telecare), monitoring (activity monitoring) pharmacological compliance (adherence to pharmacological treatment). Some systems are primarily patient care oriented; other systems are oriented to the management of clinical trials to validate a particular case study; finally, there are systems whose main purpose is to analyze the data produced and to identify significant patterns to improve care services.

The main feature of the platform is the need to flow data between the various systems in a linear, efficient and safe way. To meet these requirements, an Event Manager has adopted, whereas the information concerns creation, modification, and elimination of HHS data. The objective of the ICT research infrastructure is to enable: the integration of platforms for the provision of different services and data collection from heterogeneous sources; the collaboration of industries, research centers, healthcare facilities, public bodies on innovation objectives; the collection of information and data, their processing and the application of Intelligent Analytics and Business Analysis techniques.

### Computing infrastructure

The wide set of applications and devices benefited from large computing infrastructure, i.e., High-Performance Computing for Artificial Intelligence (HPC4AI) (Aldinucci et al. 2018). The service is offered by the University of Turin and Politecnico of Turin federated in one big cloud system, the GARR cloud, open, easily accessible by research centers and companies. This can support the experimentation of Hospital-at-home applications on a geographical scale in an immediate way. The GARR cloud, in HPC4AI, is characterized as a high-performance “AI-on-demand” platform specifically designed to support the development of ML solutions (Cerquitelli et al. 2016), BigData Analytics (BDA), with a market-specific place of solutions for engineering, natural language processing, machine vision.

Several general-purpose AI/BDA platforms and services are currently offered by commercial cloud providers as an integral part of their cloud portfolio. The majority of commercial cloud offerings have significant drawbacks concerning the need for an open AI-on-demand platform for the European research and industry:

- i. they are based on proprietary solutions that produce technological lock-in;
- ii. usage of specialized hardware at a large scale (e.g., GPU and FPGA) that are needed to address AI/BDA datasets, which are ever-increasing in size and velocity, are generally expensive. Moreover, after applications have been built, the platform will continue to exact rent for the services deployed;
- iii. the management of sensitive data (including medical data and annotated datasets) in commercial platforms is managed as a black-box with no means for the data owner to evaluate technical solutions’ effectiveness and adapt technical solutions to specific needs (e.g., enforcing specific localization of data).

HPC4AI adopts OpenStack cloud technology and extends it with federation capability (implemented by the GARR consortium in collaboration with Canonical). The solution provides an on-demand platform supporting AI and Big Data Analytics solutions as services via the GARR/OpenStack federation, managed and maintained through automated tools, greatly reducing the workforce required to implement a modern and efficient cloud computing platform. Automation is based on a declarative modeling approach that is used to describe the structure and constraints of the cloud system components. An automation tool generates a deployment plan consisting of the steps required to achieve the requested configuration, transforming the system’s state until it satisfies all constraints.

The whole project can be managed within a private instance of the Kubernetes platform running on top of a set of OpenStackVirtual Machines (VMs). This makes it possible: (a) to exploit OS containers as deployment units for several components implemented by different partners; (b) to provide elasticity to the system by way of dynamical reconfiguration of the Kubernetes deployment; (c) to enforce strong “multi-tenant” compartmentalization of computation and data by way of Openstack VMs (beyond Kubernetes namespaces); (d) to embed the whole system within the same security perimeter of the data owner, which is the University of Turin.

Besides, HPC methods and tools support the deployment of workflows by way of a novel declarative programming model supporting the definition of scientific workflows with “temporal” and “spatial” dependencies expressing respectively the pipelines of applications and software infrastructures needed to run them. No other workflow system currently supports spatial dependencies, which are typical of HPC workloads, which are required to automate the deployment of parallel and distributed applications exhibiting data dependencies, as it happens for distributed training and streaming systems (as Kafka). This workflow system also supports the precise allocation of heterogeneous resources.

### Orchestration and integration functionalities

**Fig. 2:**
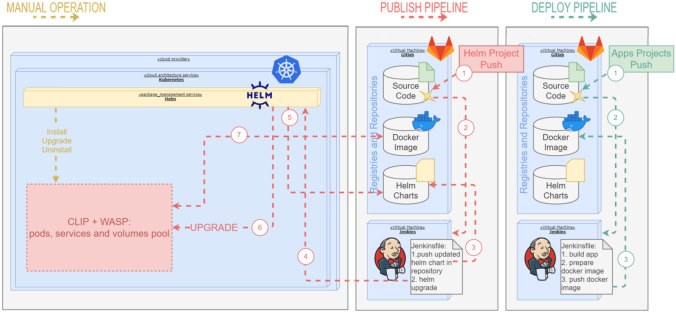
The clinical trial manager platform (CLIP)

A specific CLInical trial manager Platform (henceforth CLIP, detailed in Fig. 2) based on open-source technologies offers orchestration and integration functionalities aimed to manage one or more clinical trials. In our AAL framework, the clinical trial is described by the following steps: participant screening modes; inclusion and exclusion criteria; randomization; visits to be done during the trial; form compiling; the information related to the participant can be inserted manually by the operator can be inserted automatically through the integration of external components, such as devices or services. The access to the platform is managed by an authentication made with the OpenId Connect protocol. This authentication protocol is modular, thus can use any Identity Provider supporting it. The system currently uses an open-source application to handle the authentication, i.e., Keycloak.

The platform is built to support different configurations depending on the single-trial requirements. Thus, each trial may need the availability of different modules and services. To be able to have such freedom and easily change these configurations, we chose to proceed with the development of microservices infrastructure. This kind of infrastructure allows “remove” or “add” module back-end service without many integration and interdependence problems. Each module has its database and needs only a couple of generic modules to be completely functional.

We integrated the use of Kafka to allow the sharing of data that flows in the platform with potential external parties, being able at the same time to filter what can be shared and what not. To be environment problem-free, we run each application (back or front end) in single docker containers, which are deployed in Kubernetes. This choice allows the platform to require just the installation of Kubernetes in whatever cloud-service provider and in whatever operative system. Also adding the development of automatic deployment and publishing pipeline with the use of Jenking and Helm, we can provide a full-stack DevOps pipeline, from the continuous integration (GitLab), continuous testing (Jenkins), and code review (SonarCube), and continuous delivery (Jenking + Helm). The open-source technologies include Kubernetes, Helm, Jenkins, Docker, Keycloak, Kafka + Zookeeper, Mule ESB, Spring Boot, Node.js, React.js, Redux.js.

#### Data Ingestion platform

The data ingestion platform leverages WASP,^4^ an open-source processing framework for big data applications, for storing and processing the data produced by the other components in CANP. One of the main advantages of using the framework is to facilitate the integration of big data components; for example, an abstraction is provided for streaming pipelines, and the details of interacting with the message bus and the central database are abstracted away, allowing the applications to focus on their business logic. In this application, Kafka is used as a message bus and MongoDB as a NoSQL database. The platform can handle massive volumes of heterogeneous data; the entire platform is deployed on Kubernetes to provide an easy-to-manage and scalable system.

- WASP framework consists of four main components:
- i. Master: orchestrates the other components, starting/stopping the streaming and batch processing, and is controlled using a REST API.
- ii. Consumer Streaming: deals with the streaming processing by using Spark Structured Streaming.
- iii. Consumer Batch: is used for asynchronous processing of large amounts of data with Spark for report creation and analytics.
- iv. Producers: provides the ability to gather data from external sources or generate it.

**Fig. 3:**
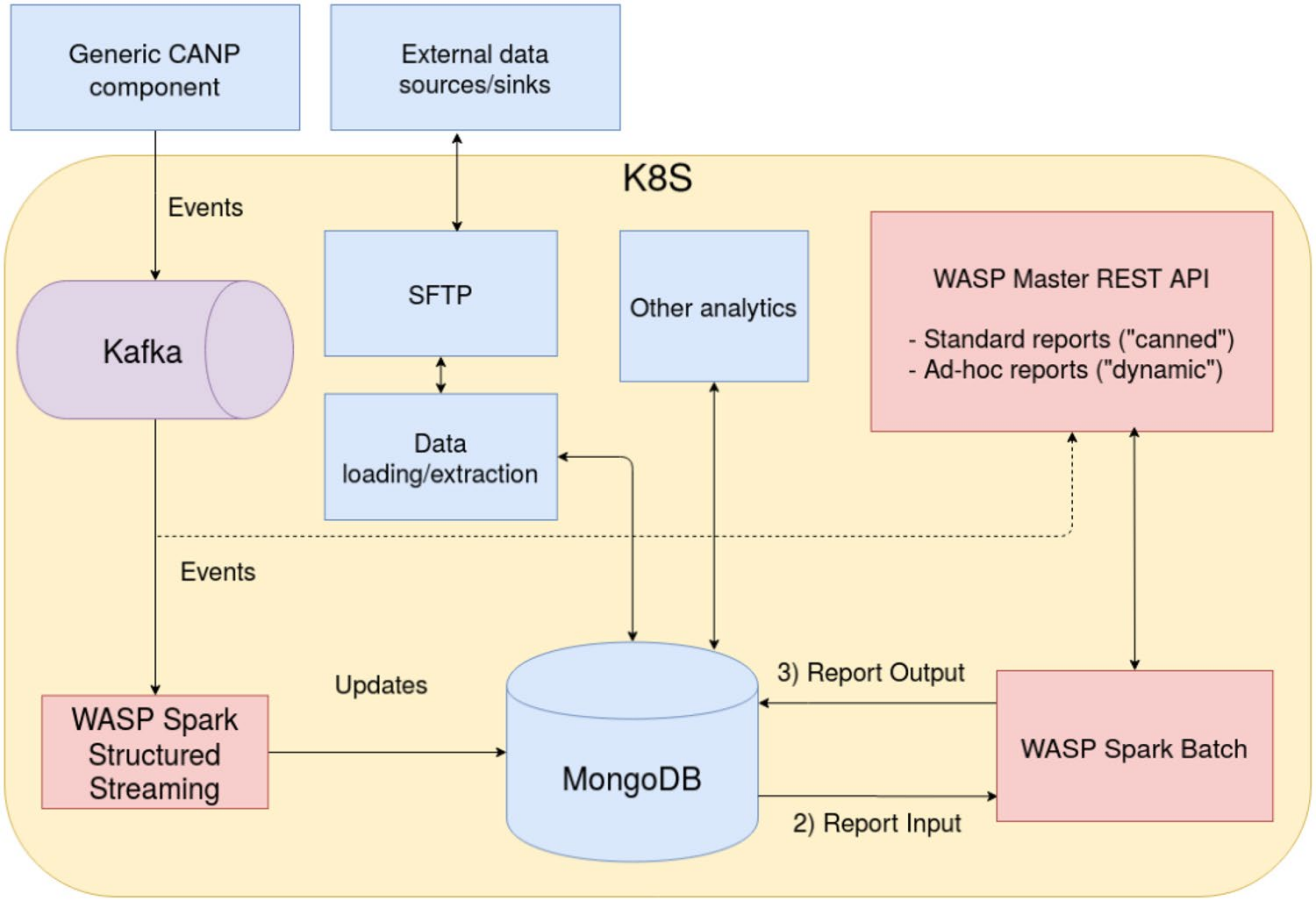
Data Ingestion framework to collect data from IoT solutions

The complete architecture is detailed in Fig. 3. The platform can receive events coming from other system components (“Generic CANP component”). They are stored and processed to generate reports or other analytics. In particular, the data flow is as follows: the various applications send data over Kafka topics as JSON messages; various topics are present, one for each data stream, to guarantee isolation and uniform schema for all the messages in a topic. This method allows the processing of them as structured data. The processing and tagging of the data in the Kafka topics are done in streaming to ensure the database’s real-time updating. The streaming processing is done with two main kinds of pipelines:

(i) Sink/Logging: these pipelines directly save the raw JSON events in a specific MongoDB collection. This allows us to keep track of every event sent and guarantees that we can retrieve it in case we need to reproduce any kind of processing by starting back from the raw data.
(ii) Main processing: these pipelines process the JSON events by identifying the data stream-specific primary key and doing any data mapping that is needed before finally inserting them onto specific MongoDB collections.

These streaming pipelines are implemented using WASP’s pipegraph abstraction and can be independently controlled by starting/stopping them using the REST API. Data stored in the collections updated by the streaming pipeline reports and other analytics generated by the batch processing component, which is triggered by the corresponding REST API. A specific module for “Data loading/extraction” is responsible for data management and importing/exporting data between the database and a staging area, in this case, an SFTP server. This allows “External data sources/sinks” to have connection points with the external world for bulk data import, report export, etc. As the staging area contains incoming/outgoing data, which is sensitive, a secure protocol was chosen to access it. Finally, other components can access the data store directly to perform “Other analytics.” One of the WASP framework’s main advantages is to facilitate the integration of big data components and ensure best practices on processes, allowing to focus on business logic.

### Stream processing platform

#### Event manager

**Fig. 4:**
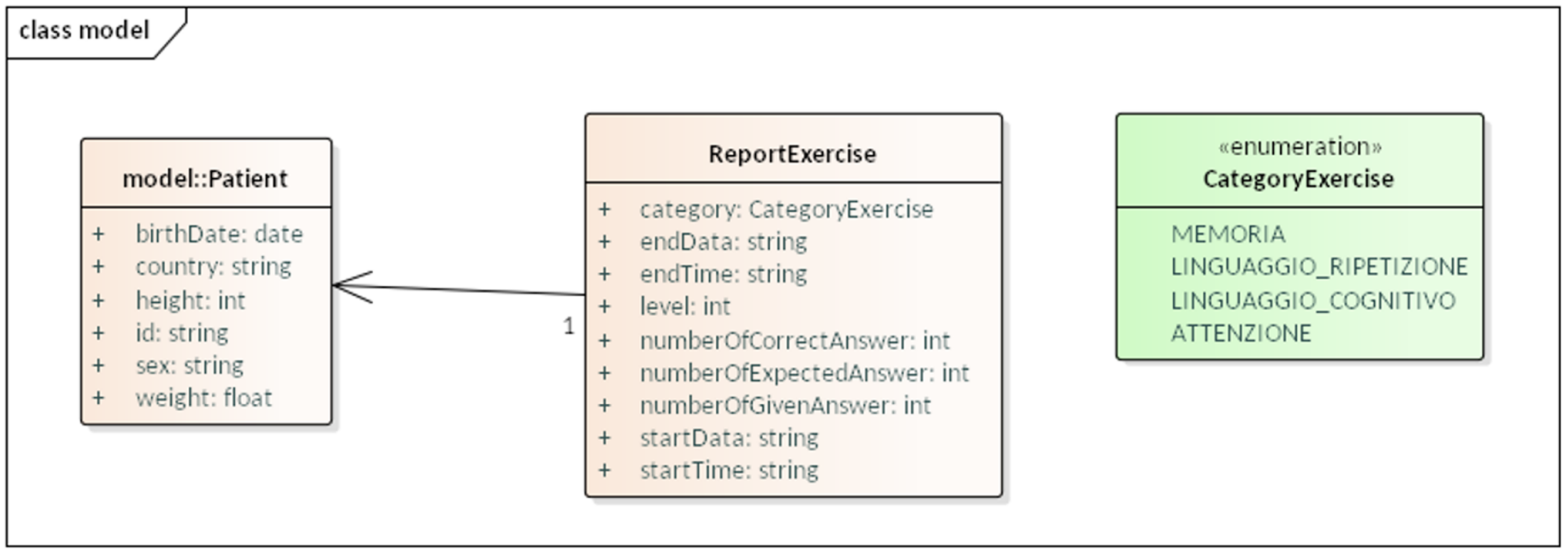
Example of how the communication between systems works. This case shows a device for language rehabilitation post-stroke

In this AAL framework, the communication of data change events occurs through a notification to the Event Manager component. The Event Manager solution allows solving two typical needs of heterogeneous systems: data exchange and systems decoupling. We observe how every healthcare system’s infrastructure can be a producer or a consumer of information about data exchange. Information producers consist of every device generating data about the corresponding activities (e.g., activity watch) and clinical data. An information consumer occurs when the information generated by the producers is useful to the Consumer System. For example, in the case of data recording for a clinical trial evaluation or in the case of statistical analysis. System decoupling concerns communication to asynchronous events, which minimizes system dependencies. All communications take place through a high-performance, highly scalable, and robust infrastructure: the Apache Kafka platform.^5^ Figure 4 describes an example of how the communication between systems works, as in the case of producer’s data (a platform for language rehabilitation post-stroke).

This system shares the most relevant data by creating a specific “topic” with a structure that contains two pieces of information: data to be shared; event type (insertion, modification, or deletion). The type of event is needed to inform consumers about the data status. This information can be useful for both Analytics and CLIP. In this case, there will be two subscribers registered on the topic that will manage the data according to their needs and timing. Data stored on the event manager remains available even if there are no subscribers at the time of registration. The event manager is a kind of transaction log that allows the consumer to move along the operations registered at different times.

The various systems of this AAL architecture are, as already mentioned, heterogeneous. Not only the functional part but also the technological part: Operating systems (Windows, Linux); Architecture (Web Application, Client-Server); Development languages (e.g., C#, C++, Java, nodeJS, angularJS). The systems, therefore, need an infrastructure that must have the characteristics of a typical Infrastructure as a Service (IaaS) but that can easily switch to a Platform as a Service (PaaS). The above-mentioned HPC4AI platform adopted in the architecture allows satisfying these different needs.

#### Assistive technologies

To provide some examples of assistive technologies exploited in this work, as a representative way of the solutions adopted at the patient’s home connected to the whole platform, we introduce here the innovative solution for elderly monitoring based upon an assistive wrist-watch, a telehealth remote care system, and a conversational agent (chatbot). We selected these three applications because they are representative of different types of assistance and monitoring, also used within clinical trials protocols: prevention (Assistive wrist-watch), enhancement of effectiveness towards caregivers (Conversational agent), and towards the patient (Teleassistance).

*Assistive wrist-watch* This device is a clock that, through several sensors, is able to record measurements relative to the movement of the person wearing it Sulis et al. (2019b). The values recorded are stored in appropriate counters, e.g. number of steps, physical activity levels. A relevant effort concerns the device integration of wrist-watch with the above-mentioned CLIP platform. The device acquires patient activity data and transmits them to a base station installed at home and connected to the mobile network. The information feed processing algorithms estimate related frailty parameters, such as mean walking speed or activity variability. A communication protocol has been designed to send counters via the mobile network at each preset time interval. The communication is carried out according to an encrypted protocol that safeguards both the reading and the communication, the inalterability of the data itself, verified by an appropriate Cyclic Redundancy Check code (CRC). The physical activity data is represented by a JSON structure, having nice readability of the data and size in terms of bytes used. In particular, communication takes place using the TCP protocol on a specific port before sending CRC is generated, and encryption is carried out. The receiving service decrypts and checks that the CRC matches. The received data is recorded on a MySql relational database. An identifier called “senderId” on the data uniquely identifies the clock that sent the data. The device service allows patients to associate a clock, then a specific “senderId,” to a user. In this way, the information “senderId” + “timestamp” of the data can trace back to the user to which the watch refers. Teleassistance: connecting different health care facilities to implement care and treatment services. The project applies a telemedicine system that in a simple and effective way allows patients (and their caregivers) to network different health facilities, even distant from each other, to realize remote services of diagnosis, assistance, treatment, and training. The system includes three main components: (i) a remote station, consisting of a transportable self-supporting structure, which also contains a high-performance optical and digital zoom camera, touchscreen monitor, loudspeaker; (ii) a control station, with dedicated hardware and software components; (iii) a unit for information transmission (voice/high-quality images), based on protected protocols. The system has proven to be an excellent tool that can be used by users without any technical knowledge even during pandemic outbreak.

*Conversational agent* A chatbot provides the patients with a tool to register their meals through a simple and carefully designed conversational agent. Conversational interfaces allow humans to interact with devices using everyday life language. The current framework supported a textual chatbot to register a patient’s diary. The design of the chatbot is simple to operate through the exclusive use of buttons, and one form introduces questions. Therefore, it is manageable in case of reduced mobility, as for HHS patients. The interface presents the different types of diets assigned to the patient by the therapist. The interaction takes place as follows: the users open the interface and authenticate themselves. For privacy reasons, they log in with an “anonymous” username, that is, with credentials through which it is not possible for the chatbot provider to recognize the patients. The tool describes the amounts of food needed for an entire day. The user keeps track of his daily diet by compiling the meal into its sub-elements, also indicating the fraction consumed of the portion through the use of buttons. A specific question periodically checks for chewing and swallowing difficulties. Finally, users can automatically log out of the interface, to ensure the authentication process. Data logging occurs continuously throughout the interaction. From the clinician’s perspective, an easy-to-read format (such as a spreadsheet file) presents data extracted from individual interactions. A table shows the responses provided for each meal, the patients’ questions, and the corresponding timestamp. Dietitians access the interface through their own set of credentials. The device helps physicians have continuous patient monitoring of their clinical nutritional progress. They can intervene immediately if problems arise. Further research studies can take advantage of the corresponding dataset stored in HPC4AI.

Data processing within the platform The CLIP platform allows devices to communicate different kinds of data, storing them on a unique database. It is possible to carry out data analysis, whereas common identifiers allow the tracing of data to the corresponding identifier/patient. The platform allows the data analysts to manage: (i) different clinical trials at the same time; (ii) the questionnaires/reports drawn up by the operators. Moreover, a single common data point allows cross-trial analysis. Finally, the ability to manage data from remote sources (e.g., receiving data directly from the participant’s home) allows the reduction of the sites involved and consequently of costs, making it suitable for the management of Virtual Clinical Trials. These are also called remote or decentralized trials, which are a relatively new and yet underutilized method of conducting clinical research taking full advantage of technologies such as apps, electronic monitoring devices, and online social engagement platforms.

### Preserving privacy and security

This Section describes the security and privacy aspects in the current architecture that integrates the HPC4AI cloud infrastructure with the “penDeepHealth” cloud platform we use for the collection and analysis of sensitive data, such as health-related data. Cloud services are typically organized into (at least) three models or tiers:

- *Software as a Service (SaaS)* The service provided to the user consists of access to applications running on a cloud infrastructure. The applications are accessed from various client devices. Users do not manage or control the underlying cloud infrastructure, including the network, servers, operating systems, storage, or even the capabilities of individual applications, with the possible exception of limited user-specific application configuration settings.
- *Platform as a Service (PaaS)* which instead of one or more individual programs, a software platform is run remotely, which may consist of a stack of coordinated services, programs, libraries enabling a specific class of SaaS.
- *Infrastructure as a Service (IaaS)* In addition to remote virtual resources, hardware resources, such as servers, network capacity, storage, and archive systems, are also made available. The hallmark of IaaS is that resources are instantiated on-demand or demand at the time a platform needs them.

Security and privacy affect the following major aspects: i. Analysis of security requirements; ii. Network access control; iii. User Access Management; iv. Network security management; v. Technical Vulnerability Management; vi. Operational Procedures and Responsibilities; vii. Malware protection; viii. Information transfer. The SaaS model is mostly concerned with privacy, whereas security is concerned across all cloud service models (Saas/PaaS/Iaas).

#### Service-level privacy and security

The service platform architecture (see Sect. 3.3) clearly decouples inbound and outbound data transfer from data storage and processing through a “data loading module”, acting as a staging area for data moving in and out from the platform. This module is the only way for outbound data and acts as a cleaning facility for critical data. It exhibits structured and hygienic interfaces clearly exposing the data attributes moving outside of the box, implementing the privacy-by-design principle. The data load module facility runs as a microservice in a “secure tenant”, a computation and storage zone which is exclusively accessible to project partners, insulated from the rest of the system, and under the administrative control of the project representatives. The secure tenant is deployed by way of the “OpenDeepHealth” PaaS, which is described in the next Section. Identification and authentication requirements concern that users are uniquely identified, as well as system administrators, have identities that correspond to accounts with elevated privileges. A procedure allows for the registration and deletion of users and specifically: the assignment and creation of user passwords are controlled through a formal management process; privilege assignments are periodically reviewed for applicability. Patients follow an Informed Consent procedure, as detailed later in the Section “Ethics Committee Procedures.”

At the client level, a set of firewall rules and layers limits the external exposure of the system. A dedicated VPN applies to client/server communications. Authentication takes place on an encrypted channel through digital certificates and a specific user-key/password pair. To protect data transmission, data encryption is guaranteed by SSL/TLS protocols through the use of OpenSSL libraries (SHA1 and Advanced Encryption Standard algorithm). Secure Tunneling/VPN is based on OpenVPN technology (encrypted point-to-point tunnels).

#### Infrastructure- and platform-level privacy and security

As mentioned in 3.2, the system underlying the data collection is HPC4AI, a cloud system specifically designed for research on AI. HPC4AI revolves around three modules: two insulated OpenStack cloud systems (development and production) with varying levels of flexibility and security, and an HPC module implemented with a traditional batch HPC cluster.

At the hardware level, the cloud systems are composed of more than 30 nodes (with 4 GPUs per node), 6 different storage systems (for a total of 3PB) with different levels of speed, robustness, and security. The HPC cluster is composed of 80 nodes with about 3000 cores and cabled with a high-speed network. The three modules are housed in a data center with Tier-III equivalent characteristics (fully redundant). One of the storages (Dell EMC2) implements hardware encryption and multi-tenancy, all the other storage can be encrypted using OpenStack volume software encryption feature.

HPC4AI implements all of the cloud service models described. IaaS is delivered via virtual machines made available to the user through the Openstack Cloud, the most widely used and maintained open-source cloud in the world. The IaaS is programmable via REST APIs accessible from GUI and command line. The two IaaS instances of HPC4AI are isolated for different needs. The production cloud provides high reliability and security, while the development cloud, which is completely isolated from development, allows for experimentation with new solutions at the “control & data plane” level of the IaaS, new storage, virtualization, networking, and authentication solutions. The production system also hosts IaaS/PaaS/SaaS services resulting from UNITO research, including the OpenDeepHealth PaaS, developed within the EU DeepHealth project.^6^ This set of services is in continuous evolution (continuous delivery) according to the DevOps model. The development system allows testing and security assessment of the IaaS/PaaS before going into production. It is worth mentioning that even the production platform is actually a platform for research and development of SaaS applications and services, but not IaaS/PaaS, testing of which can introduce security risks and is therefore confined to the development cloud system. The HPC4AI architecture addresses several issues, as stated in the information policy on personal data.^7^

OpenDeepHealth is a multi-tenant platform that enables the deployment of a private microservice execution system for the Kubernetes platform. Each user group (tenant) can easily set up a private instance, segregated at the network level of the Kubernetes orchestrator, and manage it independently (by defining a project administrator) through services for elasticity, authentication, protection of network (firewall). The user group delegates an administrator who can independently and easily configure their own private Kubernetes, which is totally isolated both at the network and storage level from the rest of the system. The storage system guarantees encryption and multi-tenancy at the hardware level. The OpenDeepHealth PaaS is used to run the platform architecture described in Sect. 3.3. OpenDeephealth adopts a VPN (Virtual Private Network), implemented by way of a FreeBSD pfsense module for any communication outside of the PaaS. This distribution is optimized to be used as a firewall and router and can be used very easily from the web interface. In this way, users and private certificates are created and managed by pfSense, while the OpenVPN module has the purpose of admitting incoming connections. A further advantage of OpenVPN-over-pfSense is to allow selective management of the accessible networks for each user, giving specific rules for each type of machine. The security outside HPC4AI is enforced as follows: 1. The transmission takes place through a secure channel; ii. This transmission is allowed only to authorized users. In particular, OpenVPN allows having on the server-side a light program with an excellent ability to maintain a high number of active connections without penalizing a security standard guaranteed by the use of cryptographic keys. The client-side program is easy to manage and can be installed on a variety of operating systems, such as Windows, MacOS, Linux, Android (from version 4.4), and iOS (from version 9.0). OpenVPN uses the OpenSSL library to provide data and channel encryption, the protocol it uses is not standard and is based on the TLS protocol. For user authentication, it uses pre-shared keys, certificates, or the use of username/password forms.

### Ethical questions

The ethical aspects concern the protection of the patient’s identity and dignity, the confidentiality of his personality and the protection of his personal data. The bioethical survey has verified the adequacy of technological solutions to ensure compliance with current regulations (national and European) and towards established ethical instances.

Procedures involve the collection of data in the medical field, such as a “clinical study” involving the use of AI instruments and devices. A clinical trial is an epidemiological study in which the goal is to gain knowledge about the incidence, etiology, diagnosis, and treatment of a morbid condition, or conversely, the state of health. This type of research typically collects data regarding the safety and efficacy of new drugs or devices. Clinical trials are of two main types: i. an observational study that demonstrates by observing events the possible effects of risks or protective factors on a group of people; ii. an experimental study (or randomized controlled trials) where the investigator introduces a new factor into the study, such as a drug, to analyze the effect on the population, or to evaluate the efficacy and adverse events of new therapies.

In this work, ethical issues were considered in different ways following the points: i. Ethics Committee procedures; ii. GDPR; iii. treatment impact assessment; iv. document archiving.

*Ethics Committee Procedures* The Ethics Committee ensures the protection of the rights, safety and welfare of trial subjects. The typical procedural steps are: i. Preparation of forms for submission to the Ethics Committee (EC) including clinical protocol, patient forms, study regulatory forms; ii. Discussion of the application by the EC (if necessary, the IEC may request additional specifications or additions); iii. Issuance of opinion by the EC (favorable or unfavorable opinion). The ethical concerns mainly involved the first step. The forms for the Clinical Protocol (i.e., the study design in detail with the planning of the clinical trial) include documents such as: Informed Consent with which the patient consents to participate in the proposed study; a Patient Information Sheet with the information to read and understand in order to provide or refuse consent to participation; a Personal Data Processing Form that the patient must sign in order to protect his/her privacy; a letter to the Family Physician to informs the patient’s general practitioner. The Regulatory Forms include at least these documents: a Letter of Intent of the Principal Investigator; a form certifying the absence of Conflict of Interest; Technical Documentation for devices or drugs (if necessary) to be tested; a sheet summarizing what data the architecture will collect; the consent to the processing of personal data must be signed to comply with national law (i.e., Italian Legislative Decree 196 of 2003 (Privacy Code) recently updated by the GDPR.

*General data protection regulation* Managing GDPR involves identifying the key figure in evaluating the assessment, as well as protecting the processing of personal data and complying with European and national privacy laws.

*Treatment impact assessment* DPIA helps the data controller not only comply with the requirements of the GDPR, but also certify that it has acted appropriately to ensure compliance with those requirements. In other words, DPIA is a procedure to assess and demonstrate compliance with data protection regulations. The data controller monitors the progress of the procedure in consultation with the DPO and—if the processing operations require it—by obtaining advice from industry experts, the Chief Information Security Officer and the IT manager.

*Document archiving* During the conduct of clinical trials, it is necessary to maintain a repository of essential documents, i.e., the *Trial master file* (Tmf). It also serves as a means for auditors, reviewers, and inspectors to verify whether the investigators have conducted the trial in line with both the trial protocol and applicable regulatory requirements, as well as the standards of Good Clinical Practice ICH E6. These procedures comply with the requirements of applicable legislation (Directive 2001/20/EC and Directive 2005/28/EC). The contents of the Tmf must be archived to ensure ready availability and direct access. Access to archived data and documents must be properly controlled and maintained throughout the archiving period.

Finally, it is worth noting that the above-mentioned ethical questions from the current AAL project has led to the creation of the Italian Society for the Ethics of Artificial Intelligence,^8^ having the legal headquarter in the Computer Science department of the University of Turin. The purpose of the Association is the diffusion of reflection on ethics and normative choices about AI and the coordination of academic, scientific and cultural activities related to the subject.

### Discussion

This Section proposes a brief identification of the crucial aspects of the proposed solution, especially when compared to some reference infrastructures in the field, as mentioned in Sect. 2.2. To better focus our proposed AAL architecture, we relied on some challenges identified by a recent review of IoT technology applications for AAL (Maskeliunas et al. 2019), namely Intelligence, IoT Security, and Integration.

*Intelligence*. This challenge involves not only automation in intelligent systems, but especially learning, adaptation, prediction, and decision making based on AI methods. In the CANP architecture, we mentioned the adoption of data mining, and ML techniques to improve the understanding of clinical outputs for medical staff. Another promising feature to be fully integrated into AAL systems is the adoption of PM techniques, as introduced in Sect. 4.3.

*IoT Security*. A relevant issue concerns providing security to home (and industrial) automation devices that typically do not have standard security technologies (Park et al. 2016). We have devoted a lot of attention to this topic, as mentioned in Sect. 3. However, the evaluation of security requirements is still an open research field. We mentioned the need to adopt a risk assessment from the architecture design phase as a crucial requirement for AAL systems.

*Integration*. Despite the absence of a common integration and interoperability standard, there is an increasing number of IoT devices. The challenge is the management of interconnections. AAL systems typically provide integration solutions, as detailed in Sect. 3.5.

We have mentioned in the related work section two of the most relevant examples of existing architectures: Fiware and universAAL. First of all, it should be noted that these platforms come from very large European projects, with a very broad and general purpose. In the context of a regional project, CANP architecture managed practical and operational steps, to calibrate the solutions against the specifics of a particular service. Similarly to existing architectures, the CANP framework is an infrastructure that is intentionally open to the addition of new services. An interesting feature distinguishing the current framework relates to the ability of the CANP platform to add AI and processing services on data that remains available beyond the life of the project itself. As a matter of fact, the data availability provided by the university-managed HPC infrastructure is a clear advantage to use the collected data in future analyses by researchers, subject to access requests and thanks to anonimization processes.

Finally, with respect to similar AAL and IoT solutions existing in the literature, we refer to Table IV in Berti et al. (2019). The CANP solution provides support for services such as “patient training”, “alarm monitoring” and “remote patient monitoring”. In terms of technologies, the platform also offers RFID support. Concerning IoT interoperability, the architecture described here also offers support for Bluetooth/radio technologies, using Kafka Event Manager (as mentioned in Sect. 3.4.2). These possibilities allow the study of organizational processes on real data both off-line and real-time. Health process analysis is a feature of great interest for future perspectives, for which we propose an introductory exploration in the following Section.

## Process-oriented AAL

### Process modeling in healthcare

It is increasingly relevant to provide the organization with a complete representation of its business processes. This means to investigate staff strategies, resources, technological equipment, communication infrastructures. Once data corresponding to activities are stored by adopting the above-mentioned architecture, an overall BPM perspective analysis can be applied by including workflow analysis, process engineering, process reorganization, and simulation (Braun et al. 2015; Sulis et al. 2019a). In this way, real event data collected in the AAL framework becomes a support for clinicians and decision-makers, e.g., by monitoring a wide set of Key Performance Indicators (Augusto et al. 2012).

A typical business process analysis pipeline usually starts with modeling the actual healthcare process with a high-level representation. This phase introduces questions concerning real processes, e.g., the redundancy of some activities, the existence of different management settings, the accounting systems, the unification of human resources management, etc. The As-Is modeling process starts with the first analysis of the process’s organizational components based on real data (e.g., duration of the activities, resources involved, analysis of stakeholders). Secondly, modeling the system’s functioning includes the creation of visual models of processes (i.e., process map or flowchart). The output of this phase is a model that has to be verified and validated by domain experts. The next Section introduces the business analysis of the case study.

### Business process analysis

A business process analysis of the HHS can be performed on top of data from the Hospital information system and from the patients’ house, as stored in the above-mentioned architecture. In this regard, an initial modeling effort consists of describing (e.g., in BPMN) the operation of the current system. In particular, the diagram describes in detail the organization of the tour visits of the staff (medical doctors and nurses) going to the patient’s home.

A business process analysis of HHS can be performed in addition to the data collected from the hospital’s information system and patients as stored in the above architecture. The hospital’s information system can register the main flow of events occurring in the daily healthcare work.

**Fig. 5:**
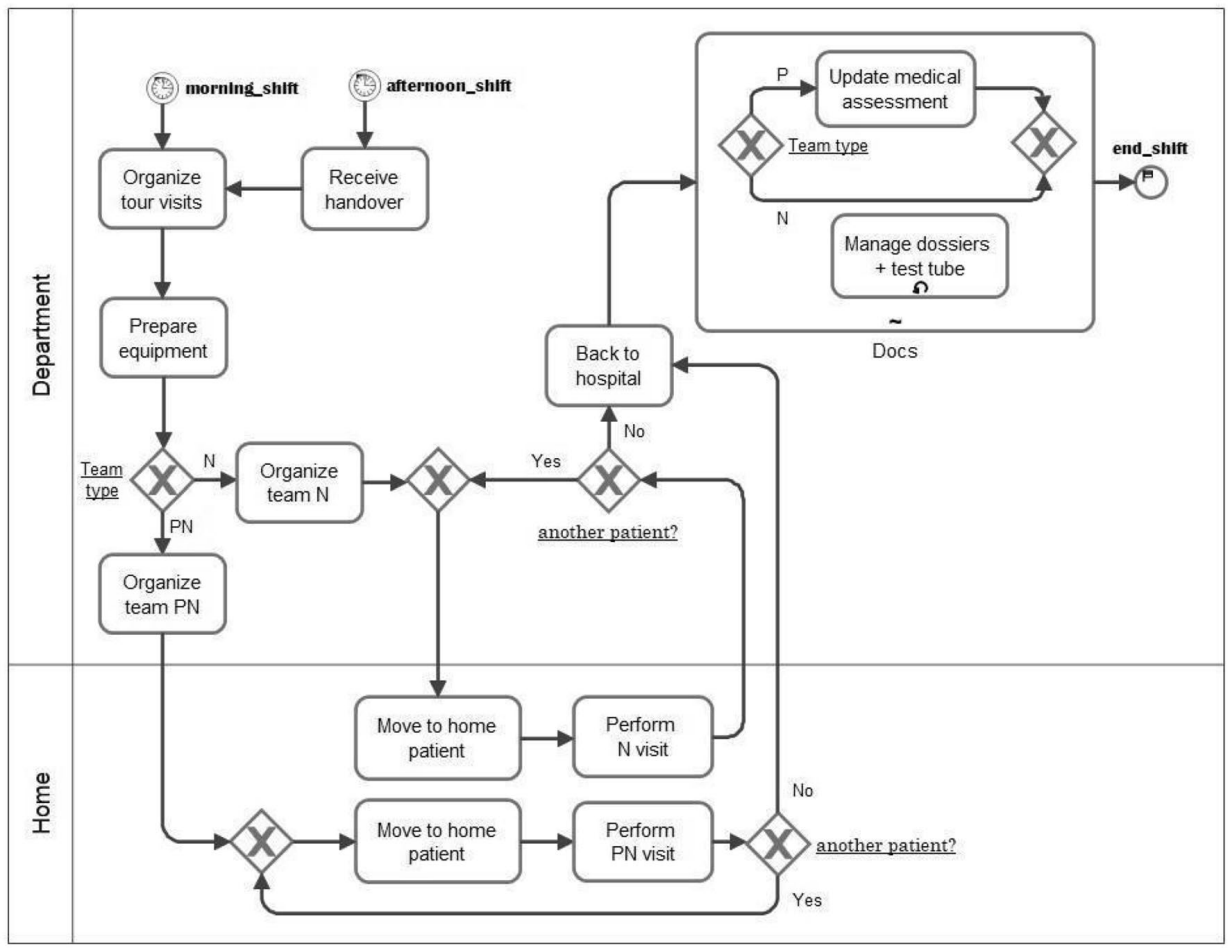
A business process model of HHS admission in BPMN

In this regard, Fig. 5 describes a BPMN model on the operation of the current system, built using traditional methods based on interviews, document analysis, observation, and so on. In particular, the diagram details the organization of staff (doctors and nurses) visits to the patient’s home. Firstly, in the morning, all the physicians and nurses together organize the tour visit (*Organize tour visits*). This activity consists of analyzing all the patient’s situations according to four impact factors: medical and nursing complexity care, condition of the caregiver, and geographical location of the house’s patient. This allows to divide the whole amount of patients in a balanced group in the sense of time to spend in visits and time to go from one to the other, and assign to each group of patients a hospital team (gateway *Team type*) composed of one physician + one nurse (*Organize team PN*) or made by only one nurse (Organize team N). Patients receive home visits every morning; some patients with special conditions (politrasfused or antibiotic therapy) may also receive an additional visit in the afternoon. Once arrived at the patient’s home *(Move to the home patient*), Hospital staff carry out the visit: only nursing visits (*Perform N)* or both physician and nursing (*Perform PN visit*). For the sake of simplicity, we grouped all the activities carried out at the patient’s home into a single activity called “visit”. It should be noted that the tasks performed are multiple and are different, both in terms of type and time and responsibility if they are carried out by a team composed of a physician and a nurse or by a nurse alone. Once the visit is finished, if there is another patient to visit (gateway *Another patient?*), the team heads to the second patient’s house. The cycle resumes until the assigned patients are not finished; only then will the team be back to the hospital (*Back to the hospital*). Thereafter, in the hospital department, both physicians and nurses have to complete some “administrative” tasks. The duties are different and specific for each of the two professional categories, including responsibility. Finally, the process model as specified by the BPMN language can be “translated” into a specification that can be driven through a workflow engine. This specification should include steps performed manually, partially automated, or fully automated by the engine with the support of the company’s IT and technology infrastructure (including the corporate network and information system).

To introduce the strategy for switching from the specification of the processes to their implementation, it is necessary a radical change of sight. It requires a shift from the workflow modeling (coordination of the processing activity) to the specification of how activities are to operate in practice and, therefore, to the modeling of the requirements that users require from the information system that must support them in their work. By moving from the process diagram (in the abstract) to its implementation, researchers have to open the “black box”, which models an activity to describe how the activity must be carried out.

### Process mining example

The general framework proposed here also considers the use of data stored on the platform as a record of process-related events. In fact, HHS modeling can benefit from information derived from the hospital’s information system, as well as from data stored in the actual platform. Process discovery explores the HHS model built from the actual data.

To provide an idea of the possibilities offered by automatic analysis of the information recorded in the AAL system, we examine a subset of data collected by the platform. In particular, the example proposed here consists of a separate module to preprocess the data and generate a DFG using PM4PY. The information considered relates to the sequence of events (coded using the standard International Statistical Classification of Diseases and Related Health Problems, ICD-9) recorded by medical staff that occurred to service patients during their stay in the service’s care.

**Fig. 6:**
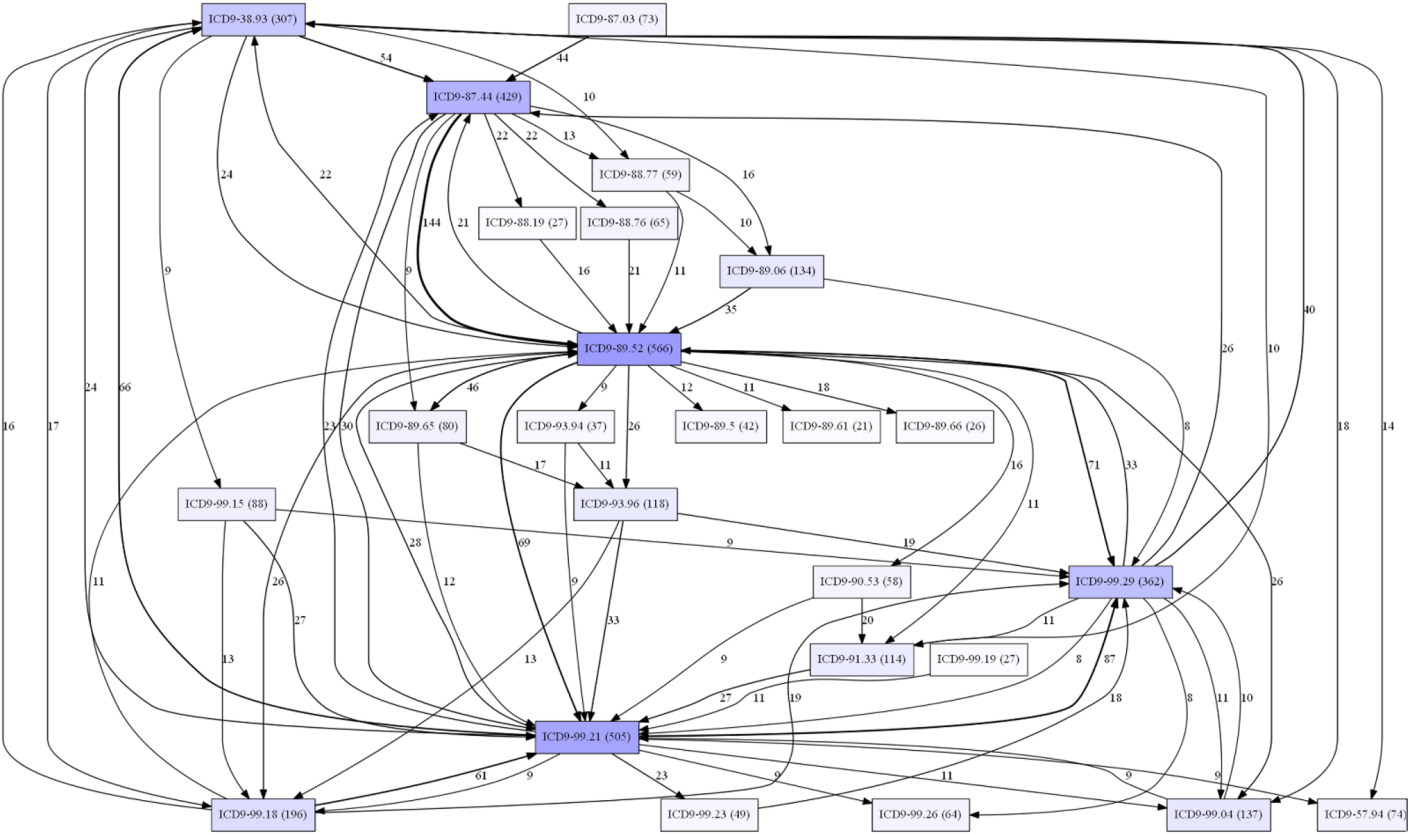
A Direct-Follow Graph based on information collected by the AAL framework. In the diagram, rectangles are events occurred to service patients during their stay in the service’s care. Each label includes the ICD-9 code and the frequency of the corresponding procedure in the considered dataset. The color highlights the most frequent events. Arcs indicate the direction of the flows. The weight of the arc is the number of connections between the respective procedures

Automatic generation of this type of process allows healthcare managers to clearly identify the most frequent pathways, as well as perform other more in-depth studies, such as business process prediction (Maggi et al. 2014) or variant analysis (Taymouri et al. 2021). For example, Fig. 6 easily identifies the main events (88.52, 99.21, 87.44) with their frequency (566, 505, 429), as well as the main flows (the weight of the arc corresponds to the number of connections between the respective procedures). Further analysis is beyond the scope of this paper, which is concerned with describing the AAL platform. However, PM techniques can add a relevant functionality to provide some insights into the role of healthcare processes, in addition to clinical concerns.

## Conclusions and future work

This work introduced an AAL framework concerning a system architecture for supporting home-care management of patients by integrating healthcare devices to assist patients at home. The AAL framework manages data from different sources. We described how to guarantee integration and interoperability. A use case focused on hospital-level care, including diagnostic and therapeutic interventions provided by healthcare professionals at the patient’s home.

We discussed how the AAL architecture impacted the following aspects: first, an improvement of professional and informal care processes, leading to a higher patient’s autonomy during acute, post-acute, and rehabilitation phases. Second, a practical solution for the integration of teleassistance/telemedicine data from home-based care and rehabilitation. Third, the investigation of different management solutions, leading to improved patient’s wellbeing and autonomy, and better and more sustainable healthcare interventions. Fourth, a reduction of healthcare and social costs. It is worth mentioning that the above-mentioned applications in the AAL system were very useful during the Covid-19 period. As a matter of fact, they allowed the continuous monitoring of patients, without exposing typically fragile people to risk infections due to visits in presence.

The current architecture opens the way to perform modeling and simulation on top of real data collected from existing systems. In particular, in future work, we plan to perform an application of PM techniques to an AAL framework, i.e., Conformance Checking, to compare the flow of the activities from the business process model and the one automatically discovered by using real data. In addition, once derived from the model, we plan to perform discrete event simulation on the top of the model to perform what-if analysis. For instance, in HHS medical process execution, a doctor may ponder whether a pharmacological therapy or a teleassistance is the best choice to be made to avoid the patient recovery; optimize the time taken for joint clinical analysis between different physicians; analyze patient’s data to obtain a more accurate report in less time; reduce the costs by optimizing the movement of hospital staff to the patients’ homes, or the drug supply chain, from management to planning, reducing waste.

Another future work is the development of a data platform to support risk management and decision support. This perspective involves predictive business process monitoring to AAL data to predict how current (uncompleted) cases will unfold up to their completion and recommend appropriate activities to healthcare managers working on patient flows. In this respect, particular attention will be devoted to older people by considering their reticence to adopt smart technologies. Previous experiences indicate the need for a caregiver to support the adoption of technological solutions.

## Data Availability

The datasets generated and analysed during the current study are not publicly available due to the agreement with hospital ethics committee but are available from the corresponding author on reasonable request.

## Footnotes

1 FIWARE architecture is the output of a project supported by the European Commission Union under the Future Internet Public Private Partnership Programme, https://ec.europa.eu/digital-single-market/en/future-internet-public-private-partnership, accessed 2022/01/10.

2 The software product is developed by the process mining group of the Fraunhofer non-profit research institute: https://pm4py.fit.fraunhofer.de, accessed 2022/01/10.

3 We refer to the CANP project, see: http://casanelparco-project.it/, accessed 2022/01/10.

4 See https://github.com/agile-lab-dev/wasp, accessed 2022/01/10.

5 https://kafka.apache.org/, accessed 2022/01/10.

6 https://deephealth-project.eu, accessed 2022/01/10.

7 https://hpc4ai.it/policy_unito/policy.html, accessed 2022/01/10.

8 The website of the newborn Italian Society for the Ethics of Artificial Intelligence: https://sipeia.diag.uniroma1.it/, accessed 2022/01/10.
